# Impact of mode of delivery on the birth experience in first-time mothers: a qualitative study

**DOI:** 10.1186/1471-2393-14-254

**Published:** 2014-08-01

**Authors:** Marie-Julia Guittier, Christine Cedraschi, Nasir Jamei, Michel Boulvain, Francis Guillemin

**Affiliations:** University of Applied Sciences Western Switzerland, Geneva, Switzerland; Department of Gynecology and Obstetrics, Geneva University Hospitals and Faculty of Medicine, Geneva, Switzerland; Lorraine & Paris Descartes University, EA 4360 Apemac, Nancy, France; Divisions of General Medical Rehabilitation, Clinical Pharmacology and Toxicology, Multidisciplinary Pain Centre, Geneva University Hospitals and Faculty of Medicine, Geneva, Switzerland; Professeure filière sage-femme, Haute Ecole de Santé, 47 Avenue de Champel, Geneva, 1206 Switzerland

**Keywords:** Mode of delivery, Delivery experience, Qualitative study, Caesarean section, Vaginal delivery

## Abstract

**Background:**

The birth of a first child is an important event in a woman’s life. Delivery psychological impacts vary depending on whether delivery has been positively or negatively experienced. Delivery experience determinants have been identified but the understanding of their expression according to the mode of delivery is poorly documented. The purpose of the study was to determine important elements associated with women’s first delivery experience according to the mode of delivery: vaginal or caesarean section.

**Methods:**

Qualitative approach using thematic content analysis of in-depth interviews conducted between 4 and 6 weeks’ postpartum, in 24 primiparous women who delivered at Geneva University Hospital in 2012.

**Results:**

Perceived control, emotions, and the first moments with the newborn are important elements for the experience of childbirth. Depending on the mode of delivery these are perceived differently, with a negative connotation in the case of caesarean section. Other elements influencing the delivery experience were identified among all participants, irrespective of the mode of delivery. They included representations, as well as the relationship with caregivers and the father in the delivery room, privacy, unexpected sensory experiences, and ownership of the maternal role. Women’s and health professionals’ representations sometimes led to a hierarchy based on the mode of delivery and use of analgesia.

**Conclusions:**

The mode of delivery directly impacts on certain key delivery experience determinants as perceived control, emotions, and the first moments with the newborn. The ability/inability of the woman to imagine a second pregnancy is a good indicator of the birth experience. Certain health professional gestures or attitudes can promote a positive delivery experience. We recommend to better prepare women during prenatal classes for the eventuality of a caesarean section delivery and to offer all women and, possibly, their partners, the opportunity to talk about the experience of childbirth during the postpartum period. The results of this study suggest that further research is required on the social representations of women and health professionals regarding the existence of a hierarchy associated with the mode of delivery.

## Background

The birth of a first child is an important event in a woman’s life [1]. A positive delivery experience can result in a sense of accomplishment and feelings of self-worth and self-confidence. However, a negative delivery experience can result in detrimental consequences ranging from feelings of maternal distress to postpartum depression and even post-traumatic stress disorder [2–4]. The infant and the partner can also experience these pathological consequences [5]. Many physical aspects have been evaluated in terms of mortality and morbidity [6], but there are much less data on the psychological aspects contributing to the construction of the delivery experience, although just as essential.

Delivery experience determinants have been identified [1, 2, 7–10], but a thorough understanding of their expression according to the mode of delivery has not been investigated to our knowledge. The mode of delivery might appear to be the most relevant predictor of delivery satisfaction [11], but it is also the most controversial variable. Historically, vaginal delivery is represented as the mode that has the best chance of being positively experienced [12, 13]. However, more recent studies suggest that elective caesarean section has higher satisfaction ratings if the maternal anxiety or stress level is taken as a reference point [14, 15]. Meanwhile, professional associations and health observers, such as the World Health Organization, warn on the increasing number of elective caesarean sections in industrialized countries, which generate supplementary costs and carry greater health risks for the mother and child [16, 17]. In the light of the debate on the increase in the number of elective caesarean sections and the medico-psychological and economic issues in terms of health policy, the influence of the mode of delivery on the construction of the delivery experience is an important aspect to consider. Hence, the aim of this study was to determine important elements associated with first delivery experience according to the mode of delivery.

## Methods

### Design

A qualitative method with in-depth interviews was chosen to explore women’s experiences with their own words and to access to their representations.

### Participants and setting

Semi-structured interviews were conducted with 24 primiparous women between 4 and 6 weeks’ postpartum from January to July 2012. The choice of the best time to collect the women’s testimony was based on results and recommendations from previous studies [18–20]. All participants delivered at the maternity unit of Geneva University Hospitals, Geneva, Switzerland.

All primiparous women delivered at term following pregnancies without major complications were eligible for study inclusion. All modes of delivery were equally represented, irrespective of the analgesic methods used. Exclusion criteria were: poor understanding and ability to communicate in the French language; maternal psychiatric disorders; age <18 years; mothers of infants hospitalised in neonatal or intensive care; and particular psychosocial situations, as single women or women in an irregular situation in Switzerland (illegal immigration), pregnancies resulting from assisted reproduction, or women attending multidisciplinary approaches to specific problems.

A “maximum variation sampling” strategy was applied to select our sample [21, 22]. This consists of deliberately choosing a sample for which large variations are expected in the dimensions of interest previously identified. We hypothesized that the mode of delivery was crucial for the delivery experience in primiparous women. Groups were thus compared according to the two modes of delivery: vaginal delivery (including equally spontaneous and instrumental vaginal delivery) and caesarean section (including equally elective and emergency caesarean section).

### Procedure

After reading the information sheet and signing the informed consent, an interview was conducted at the woman’s home (23 interviews) or on the maternity ward (one interview). Participants were questioned using standardized face-to-face semi-structured interviews [23]. Open-ended questions elicited the woman’s representations of the importance/consequences of the mode of delivery, their first delivery experience, and their views on what should be changed in midwifery accompaniment to improve a positive experience. An interview guide was developed including general topics about this first delivery experience (Table 1). The topics were not addressed in a fixed order, although the first question was always *‘What can you tell me about your first delivery experience?’* As the interview progressed, issues about the impact of mode of delivery were addressed. Two experienced researchers (MJG, NJ) not involved in patient care conducted the interviews, which lasted approximately 45 minutes. Sociodemographic and obstetrical data were collected at the end of the interview.

**Table 1:** **Interview guide**

Topics	Examples of questions
Previous representations	Before the delivery, how did you imagine this experience?
Experience	What can you tell me about your experience of this first delivery?
Emotions and associated physical feelings	How did your emotions evolve as the labour progressed?
Landmark	What were the most important aspects of your delivery experience?
Projections in the future	If we assume a second delivery, what would be your wishes now that you have had this experience?
Closing	Is there anything else you would like to add?

### Analyses

Interviews were tape-recorded and transcribed. The “MaxQDA 07” software (Maxqda 2007, http://www.maxqda.com) was used to manage and analyse the qualitative data collected. We conducted a thematic content analysis of the interviews [24]. The qualitative analysis started with individual readings by three researchers (two midwives and one psychologist [MJG, NJ, CC]). The analysis continued during data collection and the encoding process using the constant comparative method, which consists of analysing the interviews by comparing one response to earlier reported responses [25]. This analysis was subsequently used to establish analytical categories that were used as a basis for the final grid used by the three researchers to analyze the transcripts in order to maximize the theoretical sensitivity and rigour [26]. Using participant-generated data via the interviews and verifying their interpretation using triangulation between the three researchers allowed to assess their reliability [27]. Research methods to determine the credibility, conformability, and transferability were derived from previous comparable studies [28]. In accordance with the stated objectives, the analyses focused on the identification of key delivery experience elements and similarities and differences, depending on the mode of delivery. Emergent data were corroborated with existing theories and compared to previous research data to assess their degree of conformity. Finally, background data were provided to ascertain the study context and allow comparisons.

### Ethical considerations

The study protocol was approved by the ethics committee of Geneva University Hospitals (CER 11-37, matped 11-053). All participants gave written permission to perform and audio tape the interviews. The confidentiality of data was guaranteed and the results of the study are reported anonymously. Reported first names have been changed to protect the women’s anonymity.

Results’ reporting of the study adheres to RATS guidelines for qualitative research [29].

## Results

Table 2 presents the participant’s sociodemographic and obstetrical characteristics. All participants were primiparous women (mean age, 30 years); most were married and of Swiss nationality. The most represented education level was professional school. Key elements of the delivery experience included those related to the mode of delivery and others not related to it.

**Table 2:** **Sociodemographic and obstetrical data**

	Vaginal delivery (VG) n = 12	Caesarean delivery (CD) n = 12
**Age (mean-range)**	29 (20-37)	31 (22-41)
**Education**		
High school	0	2
Apprenticeship	5	2
Professional school	5	3
University	2	2
Primary school	0	3
**Marital status**		
Married	9	7
Partnership	3	5
**Nationality**		
Swiss	11	4
Other nationality	1	8
**Presentation at delivery**		
Cephalic	12	7
Podalic	0	5
**Mode of delivery**		
Spontaneous VG	6	
Instrumental VG	6	
Elective CD		6
Emergency CD		6
**Analgesia**		
Epidural or spinal anaesthesia	9	12
Meopa gas	1	0
None	2	0

### Constitutive elements of delivery experience unrelated to the mode of delivery

Elements influencing the delivery experience, regardless of the mode of delivery, were identified in all participants. They included representations and expectations, as well as the relationship with caregivers and the father in the delivery room, privacy, unexpected sensory experiences, and ownership of the maternal role.

#### Representations and expectations

Delivery representations and resulting expectations impacted on many constitutive aspects of the first delivery experience. Expectations were specifically related to the mode of delivery and medicalization; women expressed their desire to deliver naturally, with minimal interventions. A delivery hierarchy was observed in terms of representations and expectations, with vaginal delivery without analgesia being the most valued. Indeed, for some primiparous women, even if the pain was expressed as ‘*unbearable*’ , the ability to endure it played an important role in self-valorisation: *‘It’s a type of pain that’s worth being experienced’. The experience of consistency or inconsistency with expectations appeared to influence their rather positive or negative delivery experience.*

*‘I think that, if a woman delivers without epidural, she is at the top of the hierarchy, and then at the bottom when she delivers by CS’. (Karine, emergency caesarean section)*

In the spontaneous vaginal delivery group, five of six women expressed a feeling of pride, but none in the emergency caesarean section group.

*‘As I did not have an epidural, I proved to myself that I could do it. It perhaps helped me for a lot of things. I am quite proud of having been able to do it like that’. (Sophie, spontaneous vaginal delivery)*

The women’s narratives indicated that they were also confronted with the representations of the health professionals that could influence maternal choice.

*‘I can tell you that when I said “I want a caesarean section”, the midwives were shocked. It was if I had said a bad word’. (Isabelle, elective caesarean section, maternal choice)*

When expectations matched the experience, the delivery could be a rewarding opportunity to surpass one’s limits. Apart from this ideal configuration, especially in the case of emergency caesarean section, this experience could be traumatic.

*‘When I went home, I continued to cry, I wanted to be alone. I did not consider myself as a good mother. I have been frustrated because in my view, I wanted so much to give birth naturally and then I should have been able to do it. To me, it is still a failure’. (Josephine, emergency caesarean section)*

A way to overcome negative emotions resulting from an unplanned mode of delivery was to focus on the feeling of belonging to a group as a result of having just delivered.

*‘Now I am part of another group of women, those who experienced childbirth, who can talk about it. It results in identity changes’. (Nadine, emergency caesarean section)*

#### Role of the relationship with caregivers and the father during delivery

Women reported the role of the relationship with caregivers and their partner (Table 3). Participants poorly identified the role of the father at delivery. Nevertheless, four possible roles were distinguished: active or passive psychological support; witness of the event, and/or instrument. Active support and instrumentalisation were logically associated with vaginal delivery, while maternal support by the presence of the father and the “historian” role in delivery progress were equally distributed between groups.

**Table 3:** **The role of the relationship with caregivers and fathers**

	Roles of caregivers
**Inform, reassure, support**	*« I arrived a little panicked, I started to cry but there was a great team, they reassured me. » (Monica, elective CS)*
	*« The one I was looking for was the midwife. I was looking for her, she reassured me, she explained me what was happening. Frankly, I would not have experienced the delivery like this without her. » (Tania, instrumental VD)*
**Non-verbal communication**	*« They saw toward what we were being directed (CS) faster than me. Seeing their eye contacts was a bit scary. » (Karine, emergency CS)*
	**Roles of caregivers**
**Passive support by simple presence**	*« Whatever it was - the midwife or my partner - to feel supported. » (Lily, instrumental VD)*
	*« My husband was next to me, I was reassured. » (Anemone, emergency CS)*
**Active support, coaching**	*« My husband knows that I cannot breathe with strong inspirations. He told me to imagine that I was running with someone hyper-athletic. » (Natacha, instrumental VD)*
	*« He (husband) said “it is not a failure, you will not be less compared to others”. » (Charlotte, spontaneous VD with epidural)*
**Witness of the events to trace the true story thereafter**	*« Once back home, I could relive those moments that I had not realized, it is mostly him (the dad) who told me everything that had happened. » (Monica, elective CS)*
	*« I have small flashes of my delivery, but whenever I mention them, my partner explains me that it did not happen like that. » (Laura, instrumental VD)*
**Tool for women, midwives**	*« I really leaned on my husband and he counterbalanced. The midwife had shown him how to massage me, but it did not work well enough so I told him “You’re not effective”. At once, the midwife took over. The next day, I made fun of him because when he arrived he said “Ah! I have a sore leg!”. I looked at him and said “No, but wait, this is a joke, you are not the one who delivered”. In fact, I leaned hard on his leg so I think that he must have been hurt, but I told him “Well, I don”t think that it’s appropriate for you to complain”. » (Sophie, spontaneous VD without analgesia)*

CS = Caesarean section VD = Vaginal delivery.

#### Privacy

Privacy was addressed in two aspects: the “exposure” of body areas associated with sexuality that could be embarrassing for some couples, and privacy related to the number of attending persons, thus creating a different atmosphere (a cocoon or a crowded room of strangers) when the clinical situation requires that the physician needs to intervene.

*‘Before giving birth I told my husband “Most importantly, you will not go looking”. Then when the midwife told him: “come and see, sir, we can see your baby hair”, so I was slightly embarrassed’. (Laura, instrumental vaginal delivery)*

*‘I started the labor in a small room and it was good for me because I love when it’s a cocoon. I was expecting to give birth in this little cocoon and then actually after it changed completely because in the operating room, there was I do not know how many people, but as much as twenty people’. (Suzy, emergency caesarean section)*

#### Unexpected sensory experiences

Changes in sensory perceptions secondary to epidural analgesia or endorphins can be surprising. Some participants mentioned sensory perceptions potentially distressing during delivery. These perceptions were mainly related to a loss of body control, and even to a loss of mental control that raised feelings of discomfort and anxiety in some women.

*‘I felt like a head without body, as if there was nobody in there, as if I was made of plastic. It was as if I could see myself from the outside. It was a weird feeling’ (Melanie, emergency caesarean section)*

*‘I felt like being in another world, being completely in a parallel world, it was weird’. (Sophie, spontaneous vaginal delivery)*

#### Ownership of the maternal role

One fourth of all women expressed having needed time to adapt to their role of mother, and the feeling of connection to the baby occurred gradually. The absence of this feeling during the first days after delivery made women feel guilty. However, at the time of the interviews, all respondents had developed a sense of adaptation to their role of mother.

*‘I did not feel like a mother during the first days, this is what is traumatic, not the delivery!’ (Betty, emergency* caesarean section*)*

*‘I fell in love with my daughter. That was two weeks ago. It was not from the beginning, it is now’. (Suzy, instrumental vaginal delivery)*

Sometimes, the transition from “woman” to “woman and mother” was experienced as more complex. Some women felt that these two aspects could not coexist and described a kind of mourning of the woman they were before giving birth.

*‘It was a bit like the end of a life, the beginning of something unknown. In fact, I really said goodbye to the “not mum” Melanie’. (Melanie, emergency caesarean section)*

*‘I had not imagined that it would stir so many things in me, from my own childhood, how I have been educated, what education I myself would give to my child. And then I did not imagine either that it would also change my perception of my marriage. With my husband, we are no longer two, but what place do we both keep?’ (Tania, vaginal delivery)*

In some instances (both groups), the women’s narratives stressed that this change in their social identity could be accompanied by a deep emotion close to a feeling of internal disruption. In these respondents, the physical and mental transformation related to the experience of childbirth was described as an abrupt and profoundly moving, and sometimes a shocking experience.

*‘The first morning I woke up after delivery, it was strange to look at myself in the mirror and no longer have my body of pregnant woman. I was really happy the baby was there, but at the same time disoriented not to recognize myself’. (Tania, vaginal delivery)*

*‘Returning home after the birth, I was not really myself, I cried, I wanted to be alone. I did not consider myself a good mother’. (Josephine, emergency caesarean section)*

### Constitutive elements of delivery experience related to the mode of delivery

The perceived control, emotions, and the first moments with the baby are reported by women as key delivery experience elements. These are directly influenced by the mode of delivery and negatively perceived in the case of caesarean section.

#### Perceived control

A dichotomy was observed between women in the emergency caesarean section group and the others. In this group, they all reported feelings of helplessness and loss of perceived control, whereas in the other groups, some women still felt that they had control at some point during the delivery process.

*‘They did not ask me, they decided. But nobody has ever asked me: what do you think about it?’ (Nadine, emergency caesarean section)*

*‘It took me two hours to take my decision (epidural). It was accepted when I had taken the decision myself’. (Charlotte, spontaneous vaginal delivery)*

#### Emotions

According to the mode of delivery, women characterized differently their first delivery experience. In the vaginal delivery group, reported emotions were related to happiness, ‘magical moments’ , ‘most beautiful day of life’ for eight of 12 women. For the remaining four women, feelings of stress, the impression that it would never end, the fear of suction cup-induced aesthetic effects on the baby’s head, the unbearable pain resulting in loss of self-control, and the lack of confidence in the medical team dominated.

*‘There are plenty of women who say: Ah, the delivery day was the most beautiful day of my life. I do not’. (Sophie, spontaneous vaginal delivery)*

*‘It was a huge pleasure! I never felt such a pleasure. I told my husband: I feel absolutely ecstatic’. (Charlotte, spontaneous vaginal delivery)*

In the caesarean section group, emotions reported were related to anxiety, fear, disappointment, or feelings of failure for 10 of 12 women.

*‘If I except Vanina’s arrival, it has been the worst night of my life’. (Karine, emergency caesarean section)*

*‘Everything went very well, but despite this I had the feeling that it was very cold, full of light, full of noise’. (Emmanuelle, elective caesarean section)*

An adjectival dichotomy was clearly observed between women in the vaginal (*‘magnificent, wonderful, marvellous’*) and caesarean section groups (*‘cold, frustrating, anxious, stressful’*).

#### First moments with the baby

The mode of delivery influenced the first moments that women spent with their baby. In general, women in the caesarean section group felt somehow deprived of the sensory discovery of their baby (partial, or too fast vision, no touch).

*‘I just saw him above the sterile towel, but at this time I just saw the back, but I do absolutely not remember the face. To me, it was a bit hard’. (Josephine, emergency caesarean section)*

The mother-child separation is necessarily negatively experienced in the caesarean section context. Preparing the parents beforehand enables them to imagine compensations.

*‘We brought a small blanket impregnated with our odours. So he is not alone, and then he may feel a bit more comfortable too’. (Anemone, elective caesarean section)*

#### Imagining a second pregnancy

Unlike the other groups, women in the emergency caesarean section group could hardly imagine a new pregnancy.

*‘How can she (the nurse) ask me about a second delivery? Even now, I do not know if I can possibly experience it again’. (Karine, emergency caesarean section)*

## Discussion

Our findings showed that the mode of delivery influences the perceived control, the characteristics of the emotional experience, and the first moments with the newborn. These aspects revealed to be central for the construction of the delivery experience and to imagine a second pregnancy notably with detrimental aspects in the case of delivery by emergency caesarean section. The results highlighted also that women’s and health professionals’ representations played a key role in the construction of the delivery experience and sometimes led to a hierarchy based on the mode of delivery and the use of analgesia.

The impact of the mode of delivery remains highly controversial. According to Bryanton et al. [11], it is the most relevant predictor of birth satisfaction for women. The authors argue that elective caesarean section is less well experienced than the vaginal delivery attempt, emergency caesarean section included [11]. In contrast, exactly the opposite was reported by Blomquist et al. [15]. Wiklund et al. [14] reported that the scores of the personality variables were different depending on the mode of delivery. Thus, women in the vaginal delivery group increased their scores on anxiety and guilt scales, while those in the caesarean delivery group decreased their scores [14]. Another study of the same authors about maternal request for elective caesarean section reported a better birth experience for these women compared to women planning a vaginal birth (p < 0.001) [30]. For Rijnders et al. [12], instrumental vaginal delivery and emergency caesarean section can be negatively experienced, while spontaneous vaginal delivery and elective caesarean section have similar satisfaction scores. Other studies suggest a positive experience with spontaneous vaginal delivery because it is associated with a high perceived control level compared to instrumental vaginal delivery or caesarean section, and a higher feeling of accomplishment [9, 13]. These conflicting results highlight the complexity when studying the delivery experience. Most scientific investigations on the subject have been conducted using a quantitative questionnaire method. In this study, a qualitative approach was considered to be the most appropriate methodology to seek a better understanding of women’s representations about the mode of delivery. Face-to-face interviews can provide an environment in which it is easier to gain access to the women’s overall views on delivery and their way of thinking about the experience [21, 31].

Our results pointed to a significant effect of the feeling of being in control that was dependent on the mode of delivery. The concept of perceived control is defined by Wallston et al. as “*the belief that one can determine one’s own internal states and behavior, influence one’s environment, and/or bring about desired outcomes. Two important dimensions of perceived control are delineated: (1) whether the object of control is located in the past or the future and (2) whether the object of control is over outcome, behavior, or process*” [32]. Perceived control is inevitably highly subjective. In addition, this perceived control was not static during labour and delivery and evolved according to the individual event sequence and response [2, 18, 33]. Indeed, women delivering by emergency caesarean section had the perception that everything was out of control, including the delivery and the first encounter with the baby. In this group, all women had a negative delivery experience, largely explaining their difficulties to imagine a second pregnancy. These women still described a feeling of emotional vulnerability six weeks after delivery including feelings of failure, regret, and disappointment. The mode of delivery clearly impacted on the first discovery moments of the baby. With vaginal delivery, mothers reported the importance of the sensory discovery of their newborn, while with caesarean section, they insisted on the frustration related to the absence of this sensory encounter [34].

The delivery-related pain experience is a complex phenomenon that can have a negative impact, but can also result in feelings of accomplishment. For some women, pain is an essential component of the delivery experience and provides a meaning to the transition to motherhood [35]. Its absence can be considered as a loss of control [36]. This hypothesis has been supported by Rijnders et al. [12] and Green [37] who have shown that women who managed their pain without analgesia have higher perceived control and are more satisfied than others. We observed the same phenomenon in our sample. The prevailing feeling associated with delivery without epidural was pride. The latter is constructed micro-socially. Indeed, the tone of the pain feeling is described by Le Breton as largely dependent on the meaning ascribed to it in response to cultural indications [38]. This is why physical childbirth feelings may be absent for women who have no other choice than to deliver by caesarean section or unplanned epidural analgesia [39]. During vaginal delivery, it appears important to let the woman decide when she needs to use analgesia. In this sense, our study provides some insight into the importance of the mode of delivery and its ability to sustain a form of social desirability through the control of analgesic techniques that it allows or not.

About the influence of the delivery mode, findings of Lilja et al. [40] allow us a casual hypothesis. They report 22% of women with depressive mood at 10 days post-partum and affirm that depressive mood in women at childbirth predicts their mood and relationship with infant and partner during the first year postpartum. This symptomatology near delivery should be investigated according to the delivery mode and our findings about the perceived control, women’s emotions and first moments with their baby.

Our findings highlight the importance of representations by both women and caregivers in the construction of the delivery experience. Women view the birth experience as a critical time of self-affirmation that is essential to their psychological well-being [33], and our results emphasize that the representations of primiparous women can be idealized. More or less strong expectations derive from these representations and their (in)consistency with the reality will determine the positive or negative perception of this birth experience [8]. When the perception is negative, the risk of postpartum depression is increased [2, 41].

The women’s narratives suggested also that caregivers’ own representations influenced their attitudes and discourse with women, and therefore potentially affected the delivery experience. For example, although nearly one in three women deliver by caesarean section in Switzerland, similar to most industrialized countries, women reported that prenatal classes prepared them for vaginal delivery, but very little for the eventuality of a caesarean section. In addition, the caregivers’ personal opinion on the parental choice for mode of delivery was often reported as unwelcome, mainly for women who chose to deliver by caesarean section [42]. Taken together, our results highlight the necessity to investigate the women’s representation and expectations before delivery in order to help them to make the best possible informed choice.

Some caregiver gestures or attitudes need to be carefully reconsidered and adapted in an attempt to try to compensate for the shortcomings experienced. In particular, it appears essential to consider women delivering by emergency caesarean section as particularly vulnerable and to provide a better accompaniment during the intervention and early postpartum.

We identified a number of recommendations for clinical practice (see list below “*Recommendations for practice*”). For example, we recommend to provide all women with an opportunity to talk about the experience of childbirth, and if possible the father. Indeed, sometimes it is the father who is traumatized by seeing things he would rather not see, e.g., forceps or vacuum. Callister [43] has highlighted the importance for the mother to share the delivery experience with a professional with expertise in technical aid as a preventive and therapeutic action. By virtue of their skills both in obstetrics and psychology, midwives would appear to be the best qualified to do this.

### Recommendations for practice

**Prenatal**

1. Promote a reflection process on delivery representations among women and midwives
2. Prepare mother and father for a possible delivery by caesarean
3. Prepare women for a possible separation with their newborn so that they can implement compensating actions
4. Inform women about possible changes in sensory perceptions subsequent to epidural and endorphins during delivery

**Delivery**

1. Identify and respond as much as possible to mother and father expectations
2. Let the woman take the initiative to ask for analgesia
3. Hide instruments (forceps, suction cups, scissors) from parents
4. Learn about the parents’ wishes concerning the possible exposure of maternal genital areas
5. In the case of caesarean section, ensure that parents can see their infant’s face immediately if the newborn clinical status allows it

**Postnatal**

1. Encourage the mother-infant sensory encounter as early as possible following caesarean section: lower the sterile towel so that the newborn can be seen entirely by the parents, establish a ‘skin-to-skin’ as soon as possible
2. In the case of caesarean section, offer fathers the possibility to perform the symbolic gesture of ‘cutting the cord’ during newborn care, and to do a ‘skin-to-skin’ with their infant when the mother is in the operating room
3. Create a postnatal discussion space with a specialized midwife to discuss delivery progress if necessary
4. Restore a feeling of being in control to women who did not feel it during the delivery from early postpartum
5. Pay particular attention to women who deliver by emergency caesarean section

Although our findings offer insight into the understanding of the construction of the delivery experience according to the mode of delivery, the limitations of its design and outcomes should be recognized. But the most notable is the common challenge of generalizing the results to other women or to other settings with qualitative methodology. It would be interesting to further investigate the role of delivery mode for the three identified components, the feeling of control, emotions and first moments with the newborn, in a larger sample with quantitative approach.

Indirectly through mother reporting, we have highlighted different roles that can be endorsed by fathers. They appear to adapt to the requests of their wife and the midwife, but the mode of delivery necessarily impacts on their role. Some women explained that their partner negatively experienced their presence at delivery, especially when it was instrumented. Their experiences should be collected in future qualitative researches for a better understanding of their experience and the possible psychological consequences [44]. The citation of a participant perfectly illustrates this issue: *‘I think that there are not enough things for the dad, to allow him to talk about it. As the mum, we are made a fuss of, the one who gave birth, the one who can talk about it. The dad has just these mental images and it is very rare that somebody asks him “what was your experience?’ That’s also a study subject… » (Tania, instrumental vaginal delivery)*

## Conclusions

The perceived control, emotions, and the first moments with the baby are important components of the delivery experience that seem to be dependent on the mode of delivery. Certain caregiver gestures or attitudes toward the mother need to be reassessed in an effort to try and compensate for any shortcomings experienced. The (im)possibility to imagine a second pregnancy is a good indicator of the nature of the emotional and physical experience. We recommend offering all women the opportunity to talk about their experience of the delivery in the postpartum period. The results of this study suggest that further research is required on the social representations of women and health professionals regarding the existence of a hierarchy associated with the mode of delivery. The role of the father in the delivery room and an evaluation of his delivery experience need also to be considered.
